# Supplementary material for: Mottness versus unit-cell doubling as the driver of the insulating state in 1T-TaS2
Source: Nat Commun. 2020 May 18;11:2477. doi: 10.1038/s41467-020-16132-9 (PMC7235044; doi:10.1038/s41467-020-16132-9)
Supplement: Supplementary file 1 — Supplementary Information [file 41467_2020_16132_MOESM1_ESM.pdf]

Supplementary Information for "Mottness versus unit-cell doubling as the driver of the insulating state in 1*T*-TaS_2"

C. J. Butler,[1,*] M. Yoshida,[1] T. Hanaguri,[1,†] and Y. Iwasa[1,2]

[1] *RIKEN Center for Emergent Matter Science, 2-1 Hirosawa, Wako, Saitama 351-0198, Japan*

[2] *Quantum-Phase Electronics Center and Department of Applied Physics, The University of Tokyo, 7-3-1 Hongo, Bunkyo-ku, Tokyo 113-8656, Japan*

Supplementary Note 1: Partial disorder in the ACAC bulk stacking pattern.

The depiction of the three-dimensional pattern of charge order shown in Fig. 1c of the main work gives only a truncated description of the overall stacking pattern. In fact, the stacking vector $\mathbf{T}_\mathrm{C}$ has three symmetrically equivalent instantiations related to each other by rotations of 120° in the basal plane (for example, 2**a** + **b** + **c**, –**a** + **b** + **c**, and –**a** –

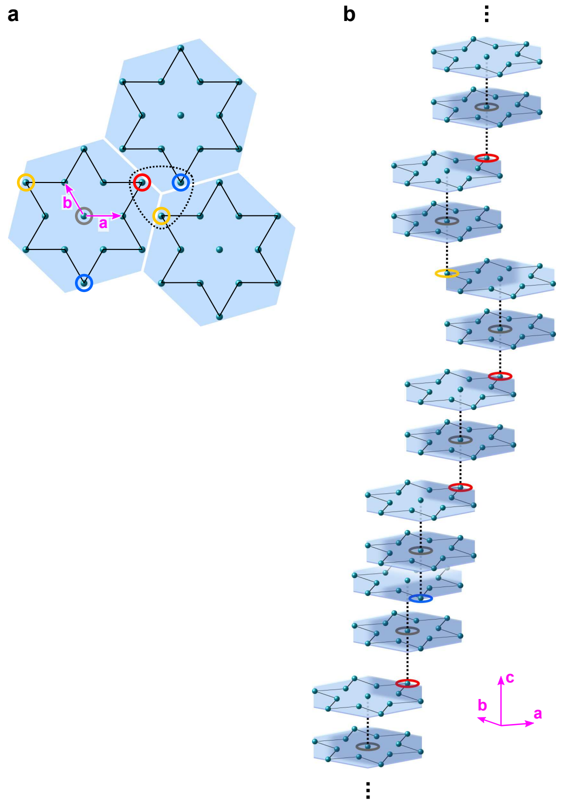

Supplementary Figure 1. **Partial disorder in the inter-layer stacking of SD cluster BLs.** **a** Three possible sites atop which successive BLs can stack. The pair of sites circled in yellow (or blue) are symmetrically equivalent through translation by one CDW superlattice vector. The sets of red, blue and yellow-circled sites are symmetrically equivalent to each other through rotations of 120° about C_3 symmetric points such as the cluster centres. **b** An extended schematic showing an example of the ACAC stacking pattern with partial disorder of the $\mathbf{T}_\mathrm{C}$ stacking vectors described previously [1, 2]. The shifted stacking between $\mathbf{T}_\mathrm{A}$–stacked BLs switches randomly between the three types of site highlighted in panel a.

* christopher.butler@riken.jp
† hanaguri@riken.jp

2**b** + **c**), and it has been established that the extended stacking pattern features a partial disorder in which $\mathbf{T}_\mathrm{C}$ varies randomly between these three. The coherence length for ordered stacking within this partially disordered pattern is ∼3 to 10 unit-cells [1–4]. A depiction of the extended stacking pattern is shown in Supplementary Figure 1. In Figs. 2 & 3 of the main work, we observe only a thin slice of the out-of-plane (OOP) stacking, and so we cannot comment on the degree of order or disorder, or the coherence length. This would require simultaneous observation of ∼10 to 100 steps and terraces – not experimentally feasible with current techniques.

Supplementary Note 2: Spatial distributions of spectral features for each of the two regular surface terminations.

In Supplementary Figure 2 below, we elucidate the spatial distributions of the prominent spectral features, which may help to understand how each corresponds to the Mott-localised orbitals, the conduction band (CB) and the CDW-reconstructed valence band (VB).

The upper panel shows that the peaks on either side of the gap for the paired surface are localised at the cluster centres, and the CDW-reconstructed bands appear as a honeycomb-like pattern around the cluster peripheries. This is consistent with the spatially resolved conductance data shown previously by Qiao *et al.* [5]. The positive spatial correlation between the spectral features at around 200 and –200 meV attests to their identification as Mott localised orbitals [6].

As can be seen in the lower panel, the spectrum for the unpaired surface shows several differences from that of the paired surface, aside from the smaller energy gap. Most notable is the pair of peaks below the gap, at around –120 meV and –240 meV. The peak residing at the higher energy, –120 meV, might be tentatively recognised as the lower Hubbard band (LHB), but if so, what is the origin of the lower lying of the two peaks? From the spatially resolved conductance at each peak energy, we see that both are localised at the cluster centres, so the lower lying of the two peaks should not be associated directly with the CDW formation or valence band. The reason is that bands which reconstruct upon formation of the SD superstructure, stabilising the CDW, are thought to originate from the twelve orbitals which lie around the periphery of each cluster, similar to the image at –450 meV [5, 7]. From the localisation of the two peaks at the cluster centres, it is reasonable that these

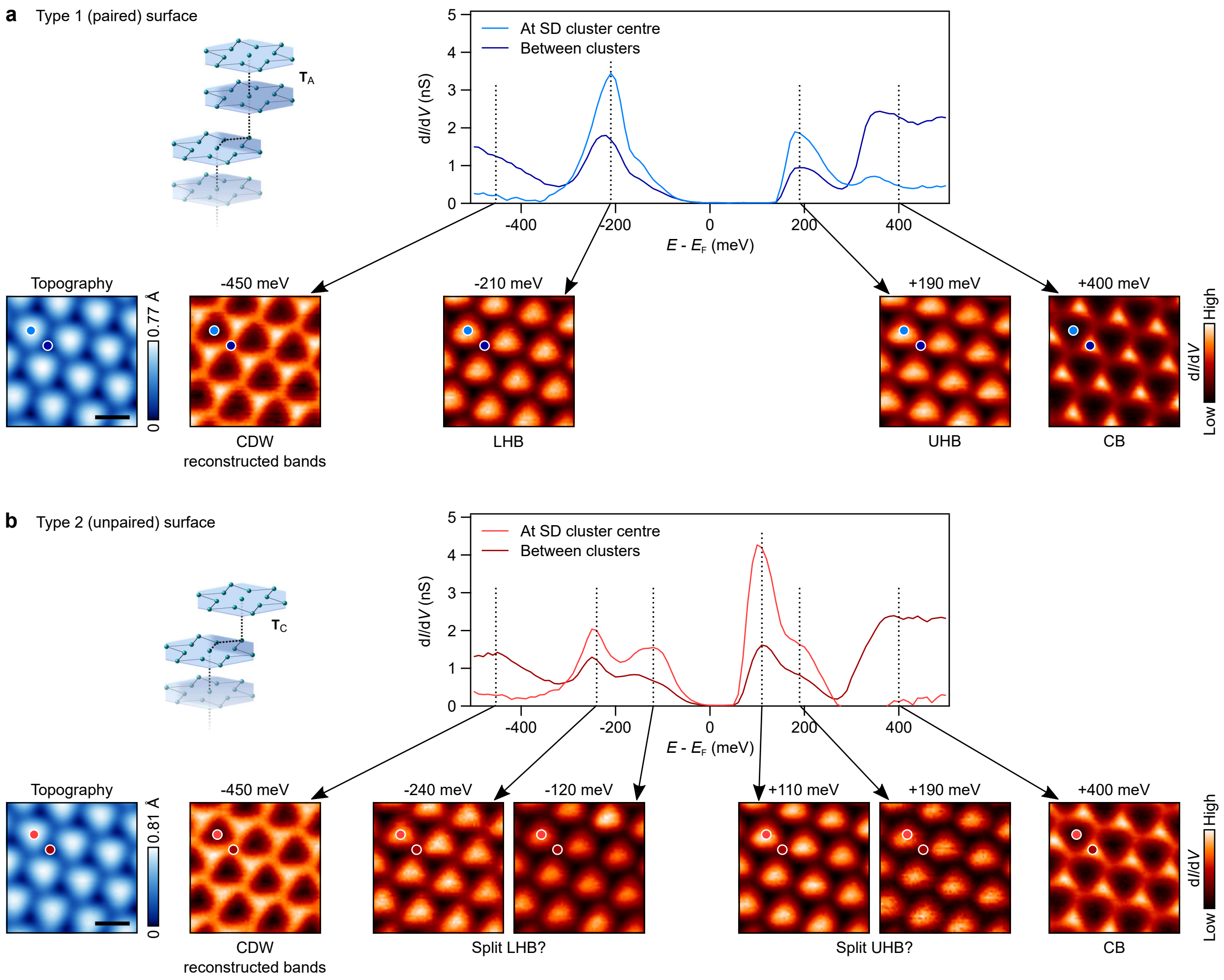

Supplementary Figure 2. Spatial distributions associated with spectral features at each of the two regular terminations. a dI/dV spectra collected at a Type 1 surface, at the centre of a typical SD cluster (light blue curve), and at the point between clusters (dark blue curve), as marked by the light and dark blue dots in the topography map. The spatial distributions of spectral features at four representative energies (marked by vertical dashed lines on the spectra) are shown in the dI/dV maps underneath. **b** The corresponding data for the Type 2 surface, acquired at the locations marked by the light and dark red dots in the respective topography image. The spatial distributions of spectral features at six representative energies are shown in the dI/dV maps. At the Type 2 surface, the first two features below E_F both appear to be localised at the cluster centres. Here, spectroscopic imaging was performed using the set-point parameters $V = -500$ mV, $I = 500$ pA, and with lock-in modulation $V_{mod} = 10$ mV. The scale bar in the topography image corresponds to 1 nm.

peaks can be described as a split LHB (and likewise for the upper Hubbard band (UHB)). However, from the observations presented here we are not able to establish the reason why the LHB or UHB may exhibit an energy splitting. We note that the spatial distributions of the CB and VB features are very similar for both surfaces, indicating that they probably reflect stacking-independent electronic structures.

Supplementary Note 3: Additional side-by-side observations of distinct terminations.

In this work, 24 surfaces were investigated and the density-of-states (DOS) spectrum with the larger gap (∼150 meV), and with the smaller gap (∼50 to 60 meV) were observed 18 and 6 times, respectively, considering only the spectrum found at the landing zone of the tip in each case. At three surfaces the type of spectrum was seen to change upon crossing some form of boundary, after moving away from the landing zone. One example, a pair of single-layer steps, is described in the main text (Figs. 2 & 3). The others, a CDW domain wall (DW), and another single-layer step, are described here.

Domain walls – in-plane phase discontinuities in the CDW – were often found at pristine cleaved surfaces. In principle, such DWs can offer opportunities to investigate the effects of inter-layer stacking: If one CDW layer with a DW is overlaid atop another layer without one, the phase jump must result in different inter-layer stacking on either side of the DW, and will be accompanied by a change in the DOS if the stacking vectors on each side are symmetrically inequivalent (e.g. $\mathbf{T}_\mathrm{A}$ versus $\mathbf{T}_\mathrm{C}$). In practice however, the same type of spectrum – either Type 1 (large gap) or Type 2 (small gap) – was almost always observed on both sides of DWs. This may be because the DWs extend downwards below the surface such that the surface layer and buried layers all have matching phase jumps, or because the change in stacking from one side to another is merely from one among the group of symmetrically equivalent $\mathbf{T}_\mathrm{C}$ vectors to another. However, on one occasion, shown in Supplementary Figure 3 below, the two types of spectrum were observable in the same field of view, in different domains separated by an unusual DW.

In Supplementary Figure 3a, ordinary DWs appear as bright linear features, along with another feature appearing as a dark line or depression (indicated by the white arrow). Here we use a partial implementation of the Lawler-Fujita algorithm [8] in order to visualise the

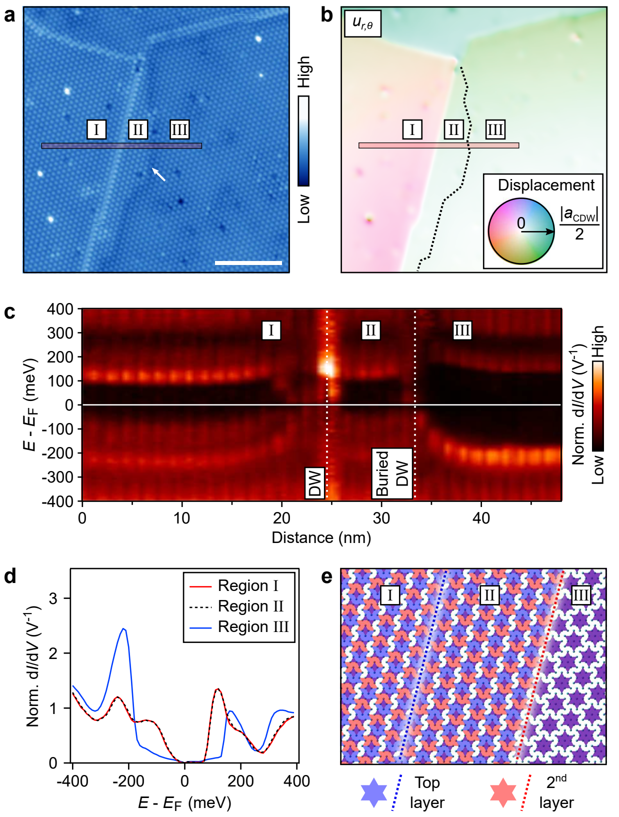

Supplementary Figure 3. Change in electronic structure with change of stacking across a buried DW. a A topographic image showing multiple DWs found at a pristine cleaved surface (V = 500 mV, I_{set} = 10 pA, scale bar 20 nm). **b** The displacement map $u_{r,\theta}(x,y)$ (with displacement taken relative to a point in the bottom-right corner) highlighting the in-plane phase jumps of the CDW pattern of the surface layer at the bright DWs. At the position of the buried DW, marked by the black dotted line, there is no displacement. **c** Spatially resolved tunnelling spectroscopy taken along the long axis of the red tinted rectangle in panels a & b, crossing both top layer DW and the buried one. A change in DOS is observed only at the buried DW. **d** Representative tunnelling spectra acquired in Regions I–III. **e** A schematic cartoon of the possible stacking configurations in each of the three regions.

in-plane phase discontinuities in the surface CDW pattern. Essentially the lock-in phase detection technique is applied to the image, yielding a map of the spatially varying phase discrepancy (displacement) between the observed CDW modulation and an ideal reference modulation. Supplementary Figure 3b shows the resulting displacement field $u_{r,\theta}(x,y)$. (The displacement is taken with reference to the bottom-right corner of the image, and expressed

in polar coordinates.) The sharp changes in hue signify in-plane phase jumps.

Importantly, although phase jumps are clear for the bright DWs, none is seen for the dark meandering feature (marked by a black dotted line). We therefore interpret this dark feature as a buried DW, with a continuous-phase layer of the CDW overlaid on top. Such a feature must be accompanied by a change of the inter-layer stacking.

As an aside, lateral strain near the domain boundaries would be expected to show up as variations in colour approaching the DWs. Instead we see only sharp transitions at the DWs, on top of smooth global variations which are attributed to hysteresis in the displacement of the STM scanning piezo-tube [9]. Therefore, it is probably not the case that interactions between tip and sample induce significant lateral strain near the DWs.

Supplementary Figure 3c shows the spatially resolved tunnelling spectroscopy collected along the red-tinted rectangle shown in Supplementary Figures 3a & b. A relatively small gap is seen on the left-hand-side (Region I), and does not change when crossing the ordinary DW into Region II. However, the spectrum does change to exhibit a large gap when crossing the buried DW in to Region III. This provides strong evidence explicitly linking the change of DOS to a change of the inter-layer stacking vector $\mathbf{T}$, although the exact stacking vectors cannot be inferred only from this measurement. Representative spectra from each of the Regions I–III are shown in Supplementary Figure 3d.

A possible scenario which could realise the above observations is shown in Supplementary Figure 3e. The top and second layer CDW patterns are depicted with blue and red star motifs, respectively. The DW in the top layer (blue dashes) changes the inter-layer stacking from one of the group of symmetrically equivalent $\mathbf{T}_{\mathrm{C}}$ vectors to another (from Region I to II). The DW in the lower layer (red dashes) changes the stacking from $\mathbf{T}_{\mathrm{C}}$ to $\mathbf{T}_{\mathrm{A}}$ (from Region II to III), which would induce the observed change in DOS from the small gap (unpaired SD layer) to the large gap (intact BL).

Supplementary Figure 4 shows a partial replication of the results in Figs. 2 & 3 accompanying the main text, acquired at the cleaved surface of a different sample. This provides supporting evidence for the distinct DOS spectra of the two terminations of the BL stacking pattern. An array of white dots (shown in Supplementary Figure 4a), along with the maps of displacement field $u_{r,\theta}(x,y)$ (Supplementary Figure 4b), show the absence of a displacement in the in-plane projected CDW pattern at the step. Within the ACAC stacking scheme, we interpret this as a step from the top of an intact BL (left-hand-side), down to the top of an

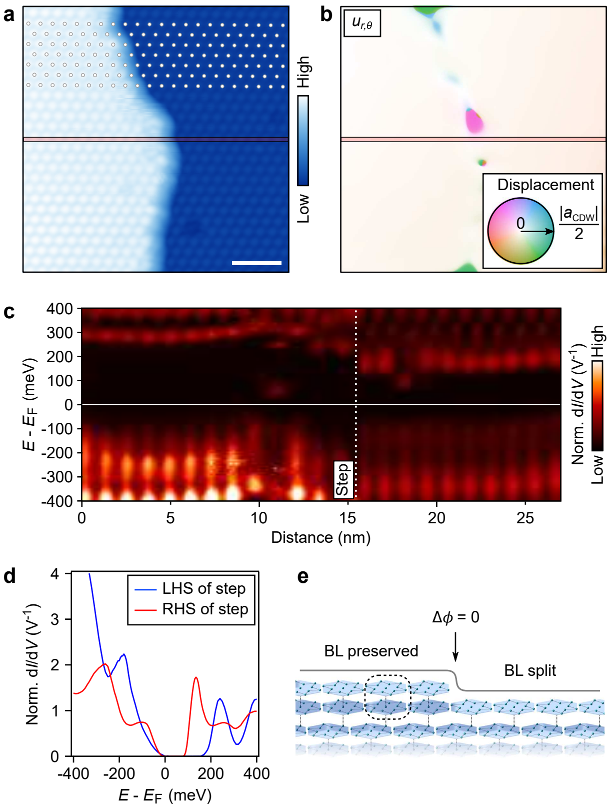

Supplementary Figure 4. Partial replication of step-terrace measurements in Figs. 2 & 3 accompanying the main text. **a** A topographic image showing two terraces separated by a single-layer step ($V = -500$ mV, $I_{\text{set}} = 500$ pA, scale bar 5 nm). **b** Map of the displacement field $u_{r,\theta}(x,y)$ (with displacement taken with respect to a reference point in the top-left corner), showing an absence of in-plane phase jump (displacement) from the upper to the lower terrace. **c** Tunnelling spectroscopy along the path marked by the red-tinted rectangle in panel a, averaged over the rectangle's short axis, and **d** typical spectra acquired on each terrace. **e** Schematic showing the correspondence between the above observations and the ACAC stacking pattern.

unpaired layer (right-hand-side), formerly the bottom half of the same BL. The concurrent change in DOS from the larger to the smaller gap, shown in Supplementary Figures 4c & d, is consistent with the findings described in the main text for Figs. 2 & 3. Supplementary Figure 4e gives a schematic showing a possible surface configuration of SD layers for this case.

Supplementary Note 4: Tip- and DW-induced band bending effects.

Tip-induced band bending – For each of the two regular types of $1T$-TaS_2 surface, the dependence of the conductance spectrum on the tip-sample separation was measured, as shown in Supplementary Figure 5. Although the absolute tip-sample separation generally cannot be known in order to compare one measurement to another, a rough proxy for it, which can be used for comparison of samples with like spectroscopic character, is the set-point tunnelling gap resistance $R_{\text{gap}} = V/I_{\text{set}}$. For the lowest tip-sample distance presented in Supplementary Figure 5 (darkest curves), $R_{\text{gap}} = 0.25$ GΩ, which is significantly lower (tip closer to sample) than for the measurements shown elsewhere in this work ($R_{\text{gap}} = 2.0$ GΩ for Fig. 2a & b, for example). From this we can safely conclude that we do not see a simple linear combination of two different DOS spectra, or that one type smoothly transforms into the other with varying tip height. The sharp conductance peaks which appear in Supplementary Figures 5c & d, at the onset of the UHB at each surface, will be discussed in detail in the future (manuscript in preparation).

The slightly larger apparent gap size in the conductance spectra here, as compared to those shown in Fig. 1e of the main manuscript, is probably due to the significantly smaller lock-in modulation amplitude used here ($V_{\text{mod}} = 1$ mV here, as opposed to 10 mV elsewhere in this work).

As both of the regularly observed surfaces (Types 1 & 2) of $1T$-TaS_2 have zero density-of-states near E_{F}, the observed spectral gap sizes may be influenced by tip-induced band-bending (TIBB) artifacts. The TIBB effect occurs due to the electric field from the STM tip penetrating into the sample. The usual outcome is that spectroscopic features are lifted to higher energies (or sunken to lower energies) while probing unoccupied (occupied) sample states. As a first approximation, the energy shifts of spectral features in the sample should monotonically follow the relative energy of the tip (with the same sign), so are still small while detecting a band onset quite near to E_{F}. We first point out that our $\mathrm{d}I/\mathrm{d}V$ curves show that at both terminations of $1T$-TaS_2, the onset of occupied states (lower edge of the Mott gap) is only 10∼20 meV below the Fermi level, so that the TIBB is very weak at the point that the onset of these occupied states is detected. Therefore, the measured energy of this lower onset should be fairly accurate.

The onset of unoccupied states does show a noticeable but weak TIBB effect. Overall,

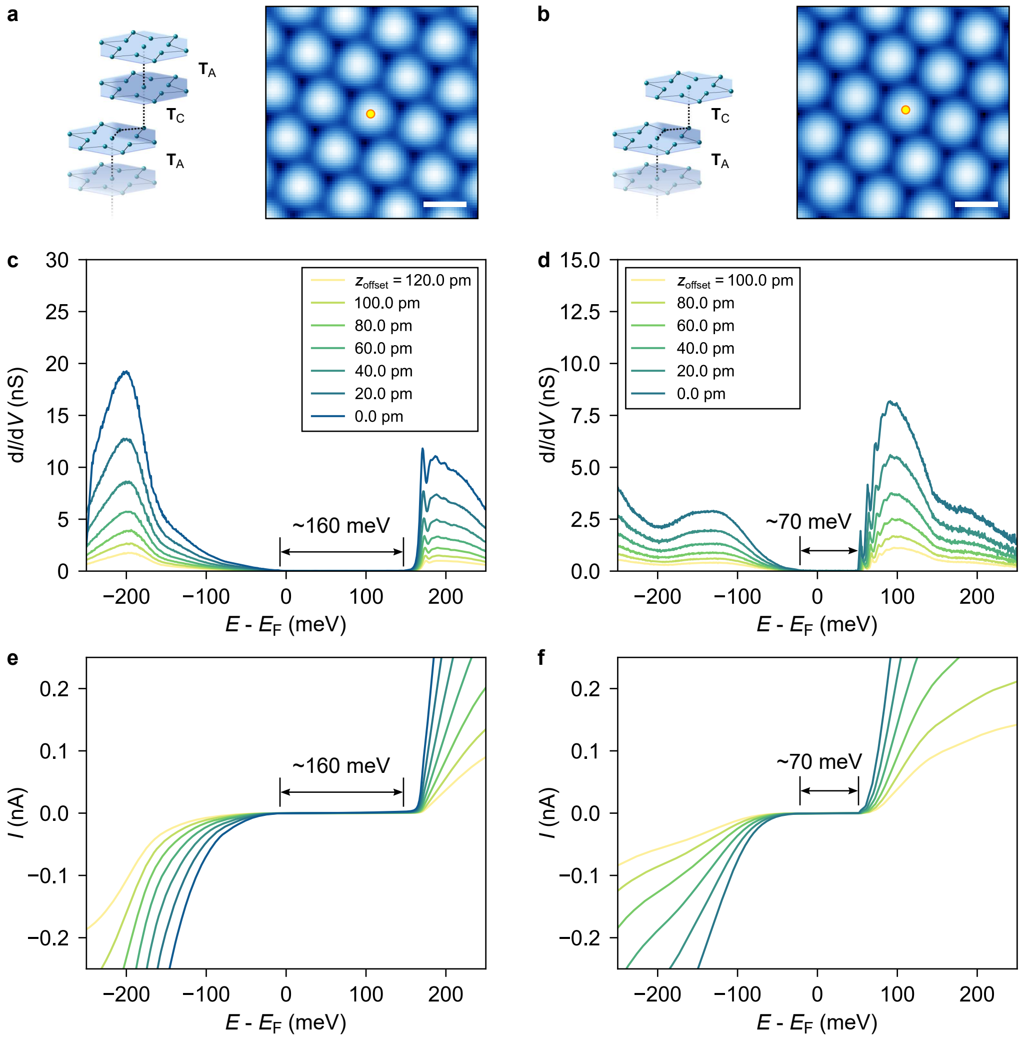

Supplementary Figure 5. Tip-height-dependence of dI/dV(V) and I(V) spectra. a & **b** STM topography maps acquired at surfaces of paired and unpaired clusters, respectively (scale bars 1 nm). Spectra were collected in the centre of SD clusters (at locations marked by the yellow dots) and sufficiently far from impurities that spectra were cluster-independent. **c** & **d** dI/dV(V) spectra obtained at $z_{\mathrm{set}} + z_{\mathrm{offset}}$. (Here z_{set} is the starting (set-point) tip height using $V = 250$ mV, $I_{\mathrm{set}} = 1.0$ nA for panel c and $V = 110$ mV, $I_{\mathrm{set}} = 440$ pA for panel d, yielding a set-point tunnel resistance $R_{\mathrm{gap}} = 0.25$ GΩ in each case.) For the dI/dV(V) curves in panels a & b V_{mod} $= 1$ mV, and therefore the apparent increase in the gap size as compared to those shown in Fig. 1e of the main manuscript is probably due to the narrower energy resolution function for the lock-in detection technique. The sharp peaks in conductance at the onset of the UHB will be discussed elsewhere. **e** & **f** The I(V) curves recorded simultaneously with the data in panels c & d.

the absence of strong tip-height-dependent energy shifts of the upper and lower band onsets indicates that these measurements reflect the intrinsic gap sizes reasonably well. Established methods such as that formulated by Feenstra *et al.* can in principle be used to account for the TIBB effect and ascertain a more accurate measurement of the gap size [12, 13]. In such a framework it is usual that in a semiconductor or insulator, a given unoccupied state ordinarily residing at E_0 should be observed at an energy E_0' which shifts increasingly higher as the tip-sample separation is decreased – i.e. $\mathrm{d}E_0'/\mathrm{d}z_{\mathrm{offset}} < 0$. For this reason it is typically thought that spectra acquired using STM overestimate the spectral gap size for a semiconductor or insulator. However, in this case there is reason for caution: Interestingly, in Supplementary Figure 5 it can be seen that in fact $\mathrm{d}E_0'/\mathrm{d}z_{\mathrm{offset}} > 0$ for the observed onsets of unoccupied states at both surfaces. Recently, Battisti *et al.* have observed that such a tendency can arise in cases where the sample work function is significantly larger than that of the tip. The apparent tip-induced potential can be non-zero even for zero applied bias, and the condition of zero tip-induced bending can be found at a non-zero applied bias, corresponding to the energy $W_0 = W_{\mathrm{sample}} - W_{\mathrm{tip}}$, where $W_{\mathrm{sample(tip)}}$ is the sample (tip) work function [14]. As in the specific case examined by Battisti *et al.*, here it is likely that a negative tip-induced potential is present for a small positive bias. (Although the sample work function can be determined, generally the tip work function cannot.) Nevertheless, Supplementary Figure 5 shows that the TIBB effect results in an energy shift of only a few meV over the range of set-point parameters typical for STM measurements. For the combination of reasons described above, we do not anticipate a large TIBB effect which would impact any of the conclusions drawn in this work.

Domain wall-induced band bending – It is well established that ordinary domain walls in $1T$-$\mathrm{TaS_2}$ can cause lateral band bending due to remaining charge density left over from partial SD clusters. It is reasonable to expect that step edges also host such remnant charge density. However, no obvious lateral band bend is seen near either of the step edges in Fig. 2b. The previously reported band bending occurred in insulating domains, whereas in Fig. 2b, the domain wall (and one of the step edges) is adjacent to the metallic domain (Region 2) into which the remnant charge density may be allowed to diffuse away. This may explain the absence of any obvious lateral band bending around the domain wall between Regions 1 and 2, and is consistent with previous observation [15]. By comparison,

the usual lateral band bending, comparable with other previous observations [16], is seen in Supplementary Figure 3c because the domains on both sides of each domain wall are insulating.

Supplementary Note 5: Supplementary data for Fig. 2.

Supplementary Figure 6 below shows the topographic line profile across the step-terrace morphology examined in the main work. The height of each step corresponds closely to the expected inter-layer spacing for $1T$-TaS_2 ($c \approx 6$ Å). The step from the large gap terrace (Region 3) up to the metallic and small gap terraces (Regions 1 & 2) appears larger than from the lower small gap terrace (Region 4) up to the large gap terrace (Region 3). A likely reason for this is the higher integrated density of states between eV and $E_{\mathrm{F,sample}}$ for the small gap spectrum, resulting in a greater tip-sample distance at these set-point parameters ($V = 250$ mV, $I_{\mathrm{set}} = 125$ pA).

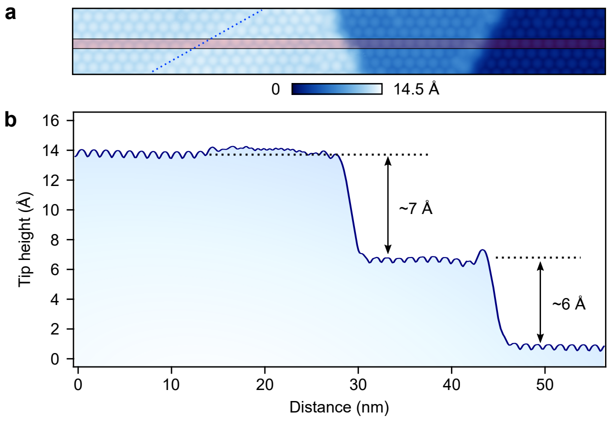

Supplementary Figure 6. **Topographic step heights of the step-terrace morphology.** **a** The same STM topograph shown in Fig. 2a of the main manuscript. **b** Apparent topographic height plotted along the long axis of the red-tinted rectangle shown in panel a (averaged over the short axis of the rectangle.)

Raw and normalised dI/dV curves corresponding to those presented in Fig. 2b of the main manuscript are shown in Supplementary Figure 7 below.

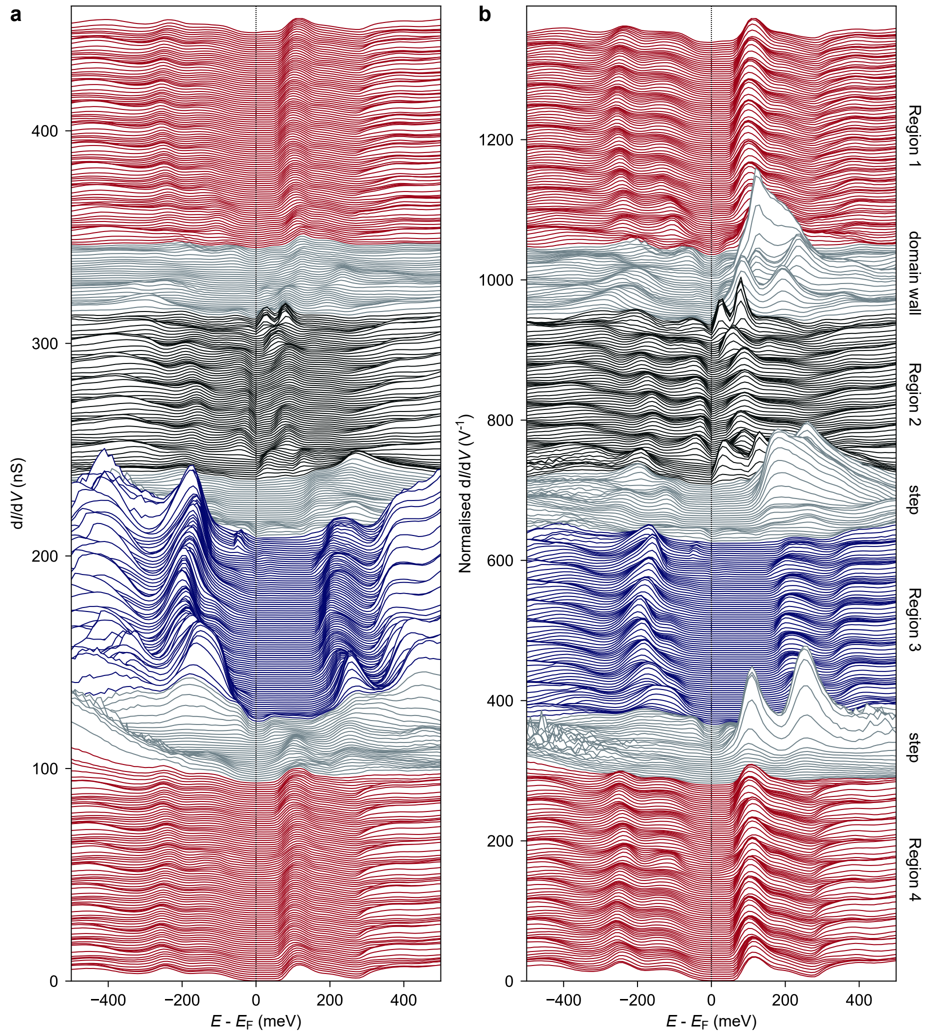

Supplementary Figure 7. **Spatial dependence of conductance spectra across the step-terrace morphology. a** Raw and **b** normalised $\mathrm{d}I/\mathrm{d}V$ curves acquired in the red-tinted rectangle in Fig. 2a of the main manuscript. The normalised data is the same as that displayed using a colourmap in Fig. 2b. (Normalisation is implemented by dividing the $\mathrm{d}I/\mathrm{d}V$ signal by the value of $I(V)$ at V = –500 mV. Curves are vertically offset by 1 nS in panel a and 3 V^{-1} in panel b.) The approximate widths of the DW and the upper and lower steps (grey curves) are 2.9 nm, 3.3 nm, and 3.4 nm, respectively, although these are overestimates due to the fact that the line-cut is not perpendicular to the DW or steps.

Supplementary Note 6: Determination of SD cluster orientation in Figs. 3c & d.

At the step-terrace formation discussed in the main text, the approximate in-plane displacements of Regions 1 & 2 with respect to the underlying BL seen in Regions 3 & 4, are

observed. But in order to interpret these displacements in terms of stacking vectors $\mathbf{T}$, the relative angular orientations of the atomic and CDW lattices must be determined.

The $\sqrt{13} \times \sqrt{13}$ $R13.9°$ superstructure of Star-of-David (SD) clusters is chiral, and can manifest in two configurations, shown in Supplementary Figures 8a & b, related to each other by a mirror symmetry operation. They can be described with angles $\pm 13.9°$ between the atomic and CDW lattice vectors.

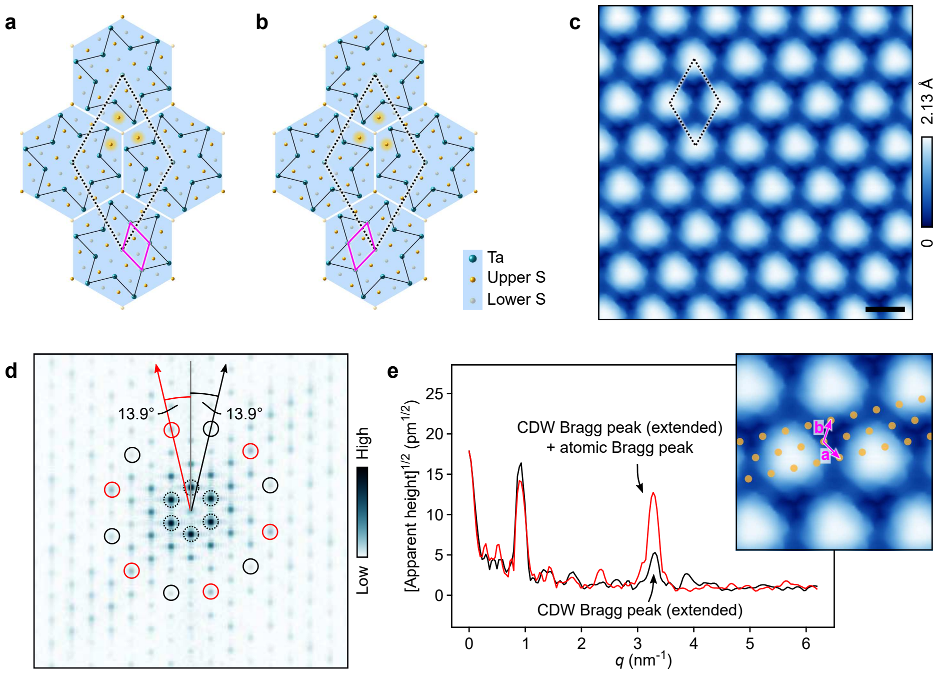

Supplementary Figure 8. Determination of relative orientations of atomic and CDW lattices. **a** & **b** Depictions of two possible orientations of the underlying atomic lattice with respect to the CDW lattice. **c** Atomically resolved STM topography acquired in Region 1 of Figs. 2 & 3 discussed in the main text ($V = 250$ mV, $I_{\mathrm{set}} = 125$ pA, scale bar 1 nm). **d** The 2D Fourier transform of the topography. First order reciprocal lattice peaks for the CDW are marked with dotted black circles. The two possible sets of locations for the atomic Bragg peaks are circled in solid black and red. **e** Line cuts through the Fourier transform at $\pm 13.9°$ with respect to the axis of the first order CDW reciprocal lattice peaks. The inset depicts the likely S surface lattice positions as yellow dots, for comparison with panel a.

Atomically resolved topography acquired in the same domain as Region 1 of Figs. 2 & 3 accompanying the main text, shown in Supplementary Figure 8c, became obtainable after the STM tip was contaminated by unstable debris. In the corresponding 2D Fourier transform

image (Supplementary Figure 8d), the first order peaks of the CDW reciprocal lattice are circled with black dotted lines. Higher order peaks of the extended CDW reciprocal lattice appear over the entire **q**-space field of view. The atomic Bragg peaks are expected to lie on top of one set of these higher order CDW peaks, at the positions marked by sets of black or red circles in Supplementary Figure 8d. Taking linecuts at $\pm 13.9°$ rotation away from the direction of a first order CDW reciprocal lattice peak (Supplementary Figure 8e), we find that the intensity at the expected position of the atomic Bragg peak is higher at $+13.9°$ than at $-13.9°$. The corresponding real-space atomic lattice is depicted in the inset, with yellow dots. The same relative orientation between the CDW and atomic lattices can be assumed for each of the three observed terraces in Figs 2 & 3 of the main work. At this point the in-plane displacements discussed in the main text can be interpreted in terms of the atomic lattice sites within the SD clusters depicted in Supplementary Figure 8a, and the stacking vectors $\mathbf{T}$ can be determined.

Supplementary Note 7: Expectation of surface metallicity in the absence of electronic correlations for the Type 2 (unpaired) termination.

We consider a simple layered system with alternating interlayer hoppings t_A and t_C, but which terminates with a vacuum beyond the top surface, as shown in Supplementary Figure 9 below. We can first imagine the case in which $t_\mathrm{A} = t_\mathrm{C}$ (as on the left-hand side of Supplementary Figure 9), which clearly corresponds to a case of half-filling in which a metallic state is realised in absence of electronic correlations. Note that this also corresponds to the purely $\mathbf{T}_\mathrm{A}$- and purely $\mathbf{T}_\mathrm{C}$-stacked $1T$-TaS_2 cases, both of which are unsurprisingly predicted, using DFT calculations without on-site repulsion U, to be metallic [2]. Now if the ratio $t_\mathrm{A}/t_\mathrm{C} > 1$ is varied, describing the case that the bulk of the structure becomes dimerised but the top layer is unpaired, we can proceed onwards to the extreme case in which $t_\mathrm{C} \ll t_\mathrm{A}$ (middle of Supplementary Figure 9), at which point it is clear that the top layer is completely decoupled, and again must be half-filled, and with an in-plane bandwidth determined by its own intra-layer hopping, which we can call $t_\parallel$. As the ratio $t_\mathrm{A}/t_\mathrm{C} > 1$ is increased, we should expect the bulk of the material to pass through a Peierls-type metal-insulator transition. But importantly, there is no point between these two limits at which the metallic state in the uppermost layer will disappear.

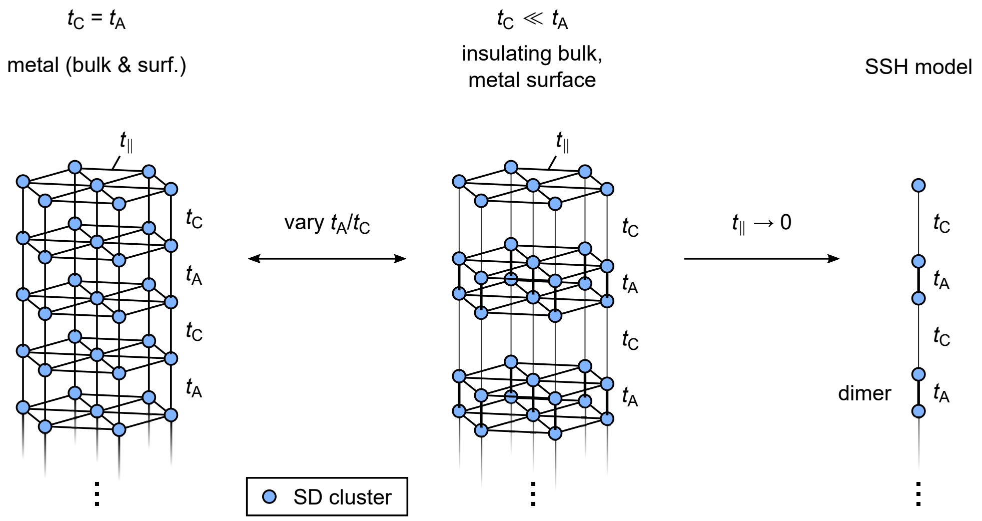

Supplementary Figure 9. **Ubiquity of surface metallicity in absence of electronic correlations for a dimerised, layered system with an unpaired top layer.** Each blue vertex represents one SD cluster. The relative distances depicted between vertices represent the relative hopping integrals between them (shorter corresponds to larger overlap).

The detailed behavior of this model is that the bulk has a gap determined by the difference in inter-layer hoppings, $|t_A| - |t_C|$, and the surface layer retains a metallic band of bandwidth $6t_\parallel$ (for a triangular lattice). Depending on the relative strengths of $t_\parallel$, t_A and t_C, the bandwidth $6t_\parallel$ may not necessarily span the dimerisation gap $2\Delta = 2(|t_A| - |t_C|)$, but a half-filled band should nevertheless persist at the surface for any set of relative strengths, as long as $t_A/t_C > 1$. (It is noteworthy that if $t_\parallel \rightarrow 0$, this model reduces to the well-known Su-Schrieffer-Heeger (SSH) model, as shown on the right-hand-side of Supplementary Figure 9, which also supports an end state at zero energy for the unpaired site.)

Does the physical system under discussion, namely the unpaired surface termination of $1T$-TaS_2, correspond to a case in which $t_A/t_C > 1$? *Ab initio* calculations performed by Lee *et al.*, indicate that the $\mathbf{T}_C$-stacked interface, specifically, should cause sufficient suppression of OOP bandwidth to open a gap overall for the bulk ACAC stacking structure [10]. This suggests that the Peierls-like dimerisation should be thought of as straddling the $\mathbf{T}_A$-stacked interface, not the $\mathbf{T}_C$-stacked interface, and therefore that $t_A > t_C$. This also matches well with the basic intuitions that (i) the larger absolute cluster-cluster distance across the $\mathbf{T}_C$-stacked interface should lead to much smaller orbital overlap, and (ii) the lateral offset across the $\mathbf{T}_C$-stacked interface should also lead to much smaller overlap due to the d_{z^2} character

of the relevant orbitals [5]. Given that the system under discussion, the unpaired surface, should correspond to the case in which $t_{\mathrm{A}}/t_{\mathrm{C}} > 1$ in the above model, this provides a strong argument that a metallic surface state is expected if electronic correlations are absent, and that such correlations are therefore the best explanation for the observed spectral gap.

Supplementary Note 8: Possible bandwidth-mediated Mottness-collapse upon change from $\mathbf{T}_{\mathrm{C}}$ to $\mathbf{T}_{\mathrm{B}}$ stacking.

Here we discuss a possible mechanism for the apparent collapse of the Mott state, leading to a metallic electronic structure, upon transition of the uppermost unpaired layer from $\mathbf{T}_{\mathrm{C}}$ to $\mathbf{T}_{\mathrm{B}}$ stacking.

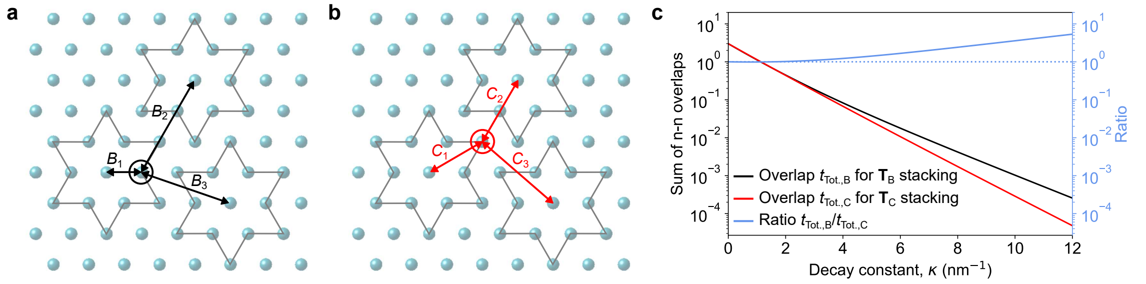

Supplementary Figure 10. Estimation of inter-layer orbital overlaps for three n-n, in an s-wave approximation, for $\mathbf{T}_{\mathrm{B}}$ and $\mathbf{T}_{\mathrm{C}}$ stacking configurations. a & **b** show the in-plane projected n-n distance hierarchies in each case. **c** shows a comparison of the respective sums of the n-n overlaps for each case, given that each n-n distance enters into an exponential decay characterised by κ. The total overlap for $\mathbf{T}_{\mathrm{B}}$ stacking always exceeds that for $\mathbf{T}_{\mathrm{C}}$ stacking in the relevant range of κ, as shown by the ratio of the sums (blue curve).

We pay attention to the stacking-dependent inter-layer orbital overlaps. As a first approximation, we may consider that the overlap integral for neighbouring orbitals is an exponentially decaying function of their absolute distance (in an s-wave approximation). Let B_{i} (C_{i}) denote the ith n-n distances for $\mathbf{T}_{\mathrm{B}}$ ($\mathbf{T}_{\mathrm{C}}$) stacking. A simple numerical calculation shown in Supplementary Figure 10, using $|\mathbf{a}| = |\mathbf{b}| = 0.34$ nm and $|\mathbf{c}| = 0.6$ nm, verifies that if all of the first three entries in the nearest-neighbour (n-n) distance hierarchy are included in a sum of exponentially decaying overlaps, then for any reasonable value of the

decay constant κ, the total overlap for $\mathbf{T}_\mathrm{B}$ stacking is always larger than that for $\mathbf{T}_\mathrm{C}$. (A reasonable value of κ means that decay length-scale $1/\kappa$ is realistically short – no more than 0.1 nm or so. In the limit of large $1/\kappa$ the total overlap tends towards the coordination number, in this case three, regardless of stacking vector.)

This simple model yields greater overlap for $\mathbf{T}_\mathrm{B}$ than for $\mathbf{T}_\mathrm{C}$, qualitatively consistent with the suggested mechanism of Mottness-collapse upon transition from $\mathbf{T}_\mathrm{C}$ to $\mathbf{T}_\mathrm{B}$.

From the idea that overlap is larger for $\mathbf{T}_\mathrm{B}$ than for $\mathbf{T}_\mathrm{C}$, it clearly follows that it would be maximised for $\mathbf{T}_\mathrm{A}$ stacking of an unpaired layer on the underlying bilayer. This leads to a tentative prediction: That such a $\mathbf{T}_\mathrm{A}$ stacked, unpaired layer atop a bilayer, yielding a top-down stacking order of AACAC$\cdots$ would be metallic at the surface due to the breakdown of the layer dimerisation (– the number of electrons per surface unit-cell is odd –) and the large inter-layer overlaps. This is consistent with the prediction of an OOP metal for purely $\mathbf{T}_\mathrm{A}$ stacking of clusters [2, 11]. However, this stacking configuration remains unobserved so far. The proposed consequences of progressively increasing inter-layer overlap are depicted in Supplementary Figure 11 below.

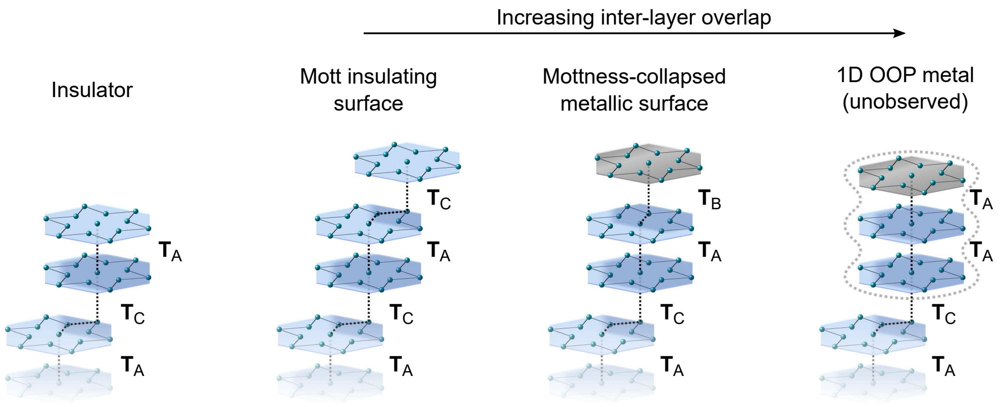

Supplementary Figure 11. **Consequences of increasing inter-layer overlaps for various surface stacking configurations.** Clusters in stacking sites which do not belong to the natural bulk stacking order are shaded in grey.

Given the simplified framework described above, the argument for Mottness-collapse at the $\mathbf{T}_\mathrm{B}$-stacked surface fits self-consistently: Inter-layer overlap, and therefore bandwidth, is expected to be larger for $\mathbf{T}_\mathrm{B}$ stacking than for $\mathbf{T}_\mathrm{C}$ stacking, potentially explaining the observed Mottness-collapse.

However, a satisfactory understanding of the mechanism of Mottness-collapse must con-

sider the hopping between orbitals centred in complex 39-atom clusters (if the top and bottom S layers are included, which have been neglected in the discussion so far), each with intricate internal bonding and orbital textures. The simplistic arguments we present above, or even explicit Slater-Koster-based tight-binding models which reduce the system only to the idealised $5d_{z^2}$ orbitals at the cluster centres, will clearly not be satisfying. A sufficiently detailed modelling of this complex system cannot be presented here, and we leave that as a target for future projects.

---

[1] Tanda, S., Sambongi, T., Tani, T. & Tanaka, S. X-Ray Study of Charge Density Wave Structure of 1T-TaS_2. *J. Phys. Soc. Jpn.* **53,** 476–479 (1984). https://doi.org/10.1143/JPSJ.53.476

[2] Ritschel, T., Berger, H. & Geck, J. Stacking-driven gap formation in layered $1T$-TaS_2. *Phys. Rev. B* **98,** 195134 (2018). https://doi.org/10.1103/PhysRevB.98.195134

[3] Nakanishi, K. & Shiba, H. Theory of Three-Dimensional Orderings of Charge-Density Waves in 1T-TaX_2 (X: S, Se). *J. Phys. Soc. Jpn.* **53,** 1103–1113 (1984). https://doi.org/10.1143/JPSJ.53.1103

[4] Ishiguro, T. & Sato, H. Electron microscopy of phase transformations in $1T$-TaS_2 *Phys. Rev. B* **44,** 2046–2060 (1991). https://doi.org/10.1103/PhysRevB.44.2046

[5] Qiao, S. *et al.* Mottness Collapse in 1T-$TaS_{2-x}Se_x$ Transition-Metal Dichalcogenide: An Interplay between Localized and Itinerant Orbitals. *Phys. Rev. X* **7,** 041054 (2017). https://doi.org/10.1103/PhysRevX.7.041054

[6] Kim, J.-J., Yamaguchi, W., Hasegawa, T. and Kitazawa, K. Observation of Mott Localization Gap Using Low Temperature Scanning Tunneling Spectroscopy in Commensurate $1T$-TaS_2. *Phys. Rev. Lett.* **73,** 2103 (1994). https://doi.org/10.1103/PhysRevLett.73.2103

[7] Rossnagel, K. On the origin of charge-density waves in select layered transition-metal dichalcogenides. *J. Phys.: Condens. Matter* **23,** 213001 (2011). https://doi.org/10.1088/0953-8984/23/21/213001

[8] Lawler M. J. *et al.* Intra-Unit-Cell Electronic Nematicity of the High-T_c Copper-Oxide Pseudogap States. *Nature* **466,** 347–351 (2010). https://doi.org/10.1038/nature09169

[9] Watashige, T. *et al.* Evidence for Time-Reversal Symmetry Breaking of the Superconducting

State near Twin-Boundary Interfaces in FeSe Revealed by Scanning Tunneling Spectroscopy. *Phys. Rev. X* **5,** 031022 (2015). https://doi.org/10.1103/PhysRevX.5.031022

[10] Lee, S.-H., Goh, J. S. & Cho, D. Origin of the Insulating Phase and First-Order Metal-Insulator Transition in $1T$-TaS_2. *Phys. Rev. Lett.* **122,** 106404 (2019). https://doi.org/10.1103/PhysRevLett.122.106404

[11] Darancet, P., Millis, A. J, & Marianetti, C. A. Three-dimensional metallic and two-dimensional insulating behavior in octahedral tantalum dichalcogenides. *Phys. Rev. B* **90,** 045134 (2014). https://doi.org/10.1103/PhysRevB.90.045134

[12] Feenstra, R. M. & Stroscio, J. A. Tunneling spectroscopy of the GaAs(110) surface. *J. Vac. Sci. Technol.* **5,** 923 (1987). https://doi.org/10.1116/1.583691

[13] Feenstra, R. M., Dong, Y., Semtsiv, M. P. & Masselink, W. T. Influence of tip-induced band bending on tunnelling spectra of semiconductor surfaces. *Nanotechnology* **18,** 044015 (2006). https://doi.org/10.1088/0957-4484/18/4/044015

[14] Battisti, I. *et al.* Poor electronic screening in lightly doped Mott insulators observed with scanning tunneling microscopy. *Phys. Rev. B* **95,** 235141 (2017). https://doi.org/10.1103/PhysRevB.95.235141

[15] Cho, D. *et al.* Nanoscale manipulation of the Mott insulating state coupled to charge order in $1T$-TaS_2. *Nat. Commun.* **7,** 10453 (2016). https://doi.org/10.1038/ncomms10453

[16] Cho, D. *et al.* Correlated electronic states at domain walls of a Mott-charge-density-wave insulator $1T$-TaS_2. *Nat. Commun.* **8,** 392 (2017). https://doi.org/10.1038/s41467-017-00438-2
